# Inhibitory Effects of 3,4-Dimethylpyrazole Phosphate on CH_4_ and N_2_O Emissions in Paddy Fields of Subtropical China

**DOI:** 10.3390/ijerph14101177

**Published:** 2017-10-05

**Authors:** Shan Yin, Xianxian Zhang, Zaidi Jiang, Penghua Zhu, Changsheng Li, Chunjiang Liu

**Affiliations:** 1 School of Agriculture and Biology and Research Centre for Low Carbon Agriculture, Shanghai Jiao Tong University, 800 Dongchuan Rd., Shanghai 200240, China; yinshan@sjtu.edu.cn (S.Y.); xixizi01090@163.com (X.Z.); jzd930303@sjtu.edu.cn (Z.J.); penghuazhu@hotmail.com (P.Z.); cs_li_98@yahoo.com (C.L.); 2 Shanghai Urban Forest Research Station, State Forestry Administration, Shanghai 200240, China; 3 Eco-Environmental Protection Research Institute, Shanghai Academy of Agricultural Sciences, 1000 Jinqi Road, Shanghai 201403, China; 4 Key Laboratory for Urban Agriculture, Ministry of Agriculture, 800 Dongchuan Rd., Shanghai 200240, China

**Keywords:** DMPP, paddy field, urea fertilizer, greenhouse gas emission

## Abstract

3,4-Dimethylpyrazole phosphate (DMPP) has been widely employed to reduce nitrogen leaching and greenhouse gas emissions in the soils of dry farmlands. However, the effects of DMPP on the dynamics of nitrogen in paddy fields remain unclear. For this study, treatments with 0%, 0.25%, 0.5%, 1%, or 1.5% DMPP levels of nitrogen fertilization plus urea were designed to determine the effects on greenhouse gas emissions in paddy fields of subtropical China. All DMPP treatments significantly reduced CH_4_ and N_2_O emissions, from 54% to 34%, and 94% to 39%, respectively, compared with a urea fertilizer treatment alone. The soil NH_4_^+^ content decreased and NO_3_^−^ increased more slowly with the application of DMPP. The crop yields under the various DMPP treatments showed no significant difference (*p* < 0.05). We concluded that the application of 0.5% and 1% DMPP may significantly reduce CH_4_ and N_2_O emissions in contrast to other treatments. This has important implications for the maintenance of rice yields, while reducing greenhouse gas emissions in paddy fields.

## 1. Introduction

The application of nitrogenous fertilizers increases crop productivity but can cause serious environmental problems. For the last 30 years, nitrogenous fertilizer consumption in China has increased by 3.28-fold; however, nitrogen use efficiency is only 30–35%, which is much lower than the average value (40–60%) at the global scale [1,2]. Increased nitrogen losses through volatilization, leaching, runoff, and denitrification/nitrification are caused by excessive fertilization. In China, nitrogenous fertilization in paddy fields has led to a series of environmental problems, such as water pollution, soil acidification, and greenhouse gas (GHG) emissions [3,4,5]. 

Numerous potential methods have been employed to enhance nitrogen utilization, while reducing the GHG emissions that are related to nitrogen fertilizer use. For instance, nitrification inhibitors (NIs) and slow-release fertilizers have been added to chemical fertilizers in order to inhibit NH_4_^+^ conversion to NO_3_^−^ [6,7,8]. As new chemical compounds that are utilized in agriculture and horticulture, NIs are effective in preventing the transformation of nitrogen to NO_3_^−^, thus they could increase both the content of ${\mathrm{NH}}_{4}^{+}–N$ and the recovery of nitrogen efficiencies over long periods [9,10,11]. The application of commonly used NIs has been considered to be an effective strategy to increase crop yields and nitrogen use efficiencies, with mean increases of 7.5% and 12.9%, respectively [12]. 

As one of the highly effective NIs [13,14], 3,4-Dimethylpyrazole phosphate (DMPP) exhibits highly favorable attributes for optimal nitrification inhibition and non-toxicological or ecotoxicological side effects [13,15]. At a high soil water content (>80%), denitrification is the primary source of N_2_O, and its emissions may be decreased by 23–45% with the use of DMPP [16]. The impacts of the application of DMPP have been investigated not only as it relates to nitrogen transformation [17], but also N_2_O and CH_4_ emissions from soils [18,19], NH_3_ emissions [20], and nitrifiers and denitrifiers [21] in dry lands. Several experiments have also been conducted on crop yields [19,22]. 

Rice is an important staple in many parts of the world, and is a semi-aquatic species that grows primarily under flooded lowland conditions in paddies [23]. GHG emission from rice paddies is a major contributor to agricultural emissions. Hence, it is critical to identify and develop effective measures to reduce N_2_O and CH_4_ emissions in paddy soils. In the present study, a five-level DMPP experiment was designed for a rice-bean rotation system in an alluvial plain in the northern subtropical area of Central Eastern China. Our aim was to examine the effects of DMPP application on (1) rice yields, and (2) GHG emissions in subtropical China. 

## 2. Materials and Methods

### 2.1. Experimental Site

The experimental site was located in the Experimental Farm of Shanghai Jiao Tong University (121.49° E, 31.04° N), Minhang District, Shanghai, China. The area is characterized by a humid subtropical climate according to a modified Köppen climate classification. During the experimental period of 2012–2013, the mean annual temperature and precipitation were 18.4 °C and 1242 mm, respectively. The soil was anthrosols according to FAO (Food and Agriculture Organization of the United Nations) classification, and the main properties starting from the surface down to a 10-cm depth were as follows: pH of 7.32, EC of 0.137 ms cm^−1^, total nitrogen content of 1.39 g kg^−1^, available phosphorus content of 9.45 mg kg^−1^, total carbon content of 9.37 g kg^−1^, and cation exchange capacity (CEC) of 17.5 cmol kg^−1^.

### 2.2. Experimental Design

Three plots with dimensions of 8 m × 8 m were set for each treatment. The crop rotation was rice (*Oryza Sativa* L.)/faba bean (*Vicia faba* L.)/rice (June–October for rice, and November–May for beans). 

The field experiment began in 2012 with different concentrations of the DMPP plus urea fertilizer. Urea was added to the fields at a traditional local level of 300 kg N ha^−1^ during the rice growing season. 

In 2012, DMPP with four levels of nitrogen (0%, 0.5%, 1%, and 1.5%) was applied to the paddy fields. Prior to the rice being transplanted, phosphorus and potassium fertilization was conducted. Urea fertilizer alone, or urea plus DMPP fertilizer was applied early, on 28 June (day 1 following transplantation). The rice was harvested on 26 October of the same year. All plots were regularly irrigated up to a water depth of 10 cm, except for the paddy field drying period, which proceeded from day 14 to 17, and day 105 until the harvesting period. 

Following the rice harvest, faba bean seeds were sown on 8 December, 2012, where no fertilizer was applied during the growing season. In May 2013, the faba beans were plowed into the fields without harvesting with green manure prior to rice transplantation. 

In 2013, DMPP containing five levels of nitrogen was applied (0%, 0.25%, 0.5%, 1%, and 1.5%) to the fields. Urea with DMPP was applied on 6 June 2013, during the first day of rice transplantation. Additional agricultural managements were the same as those performed in 2012.

All experimental treatments were designed following a completely random order with three replications. Different treatments used in this research were labeled as CK (urea only), 0.25% DMPP, 0.5% DMPP, 1% DMPP, and 1.5% DMPP. 

### 2.3. Measurement of CH_4_ and N_2_O Emissions and Crop Yields

GHG emissions were detected using the static chamber/GC system method [24,25]. During the day (9:00 a.m.–12:00 p.m.), gas samples were collected using syringes, transferred into 100-mL aluminum foil bags (Delin, Dalian, China), and immediately brought to the laboratory to analyze N_2_O and CH_4_ through configured gas chromatography (Agilent 6890N, Santa Clara, CA, USA). The fluxes of gases were calculated from the rate of gas concentration change during the sampling time. The calculation was as follows:

F = (*d*C/*d*t) × (mPV/ART) × (mP/RT) (1)

where (*d*C/*d*t) is acquired through the linear regression equation. The value m is the molecular weight of trace gas, P indicates the atmospheric pressure (P = 1.013 × 10^5^ Pa), R is the gas constant (R = 8.314 J mol^−1^ K^−1^), and T is the air temperature inside the chamber. V, H, and A are the volume, height, and area of the static chamber, respectively.

Before harvest, crop yields from the three selected 0.5 m × 0.5 m areas were measured for each treatment. Subsequent to drying at 105 °C, the rice biomass was weighed to calculate the crop yield.

### 2.4. Measurement of Soil NH_4_^+^ and NO_3_^−^ Concentrations

Soil samples were extracted from the plough layer (0–10 cm) once the rice was transplanted. Samples were transferred to the laboratory and stored in a refrigerator at −20 °C until they were analyzed. Inorganic N (NO_3_^−^ and NO_3_^−^) from the soil was measured on the extraction of moist field soil.

We removed inorganic N from the paddy soil by shaking 5.0 g of fresh soil into a 50-mL 2 mol L^−1^ KCl solution, which remained therein for 1 h. Samples were then centrifuged and filtered through filter paper (11 μm) to remove particulates or clays, and the filtrate samples were preserved at 4 °C prior to analysis. NH_4_^+^ and NO_3_^−^ concentrations were analyzed by colorimetric determination method using a SmartChem Discrete Auto Analyzer with a detection limit of 0.001 mg/L (SmartChem 200, Alliance, France).

### 2.5. Data Analyses

The global warming potential (GWP, kg CO_2_-eq ha^−1^ on a 100-year scale) was calculated to estimate the potential greenhouse effects of CH_4_ and N_2_O emissions. This result indicated that CH_4_ and N_2_O emissions were converted into the CO_2_ equivalents via the following equation:

(2) $$\mathrm{GWP}=21\times E_{{\mathrm{CH}}_{4}}+310\times E_{N_{2}O}$$

where $E_{{\mathrm{CH}}_{4}}$ and $E_{N_{2}O}$ are the accumulated CH_4_ and N_2_O emissions during the rice growing season, respectively. These were used to estimate the potential greenhouse effects of CH_4_ and N_2_O emissions by converting them into their CO_2_ equivalents.

The index of yield-scaled CO_2_-eq (GWPI, kg CO_2_-eq kg^−1^ yield) was calculated to indicate the amount of GHG that was consumed during the rice growing period, and to evaluate the environmental effects on crop output. The equation of GWPI is as follows:

GWPI = GWP/Yield(3)

All statistical analyses were conducted using OriginPro 8.5.1 (Systat Software Inc., San Jose, CA, USA) and SPSS16.0 (IBM Co., Armonk, NY, USA). 

## 3. Results

### 3.1. Methane Emissions

During the rice growing season, the CH_4_ emissions under all DMPP treatments were significantly reduced (*p* < 0.05) compared with the values under the CK treatment (Table 1). CH_4_ emissions decreased by 33.5–53.9% and 3–94% following the application of DMPP in 2012 and 2013, respectively (Table 1).

**Figure 1:**
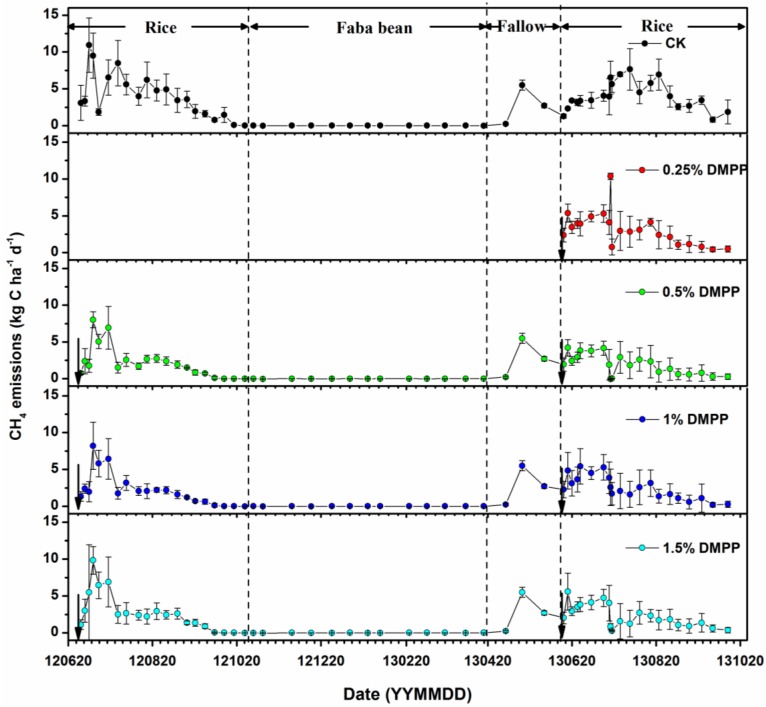
CH_4_ emissions under different DMPP treatments.

During the period of continuous flooding, the CH_4_ emissions gradually increased; however, they rapidly dropped to almost zero after a few days, due to midseason aeration. The CH_4_ emissions increased again subsequent to re-flooding. A strong seasonal variation was characterized by two pronounced higher values. The first peak occurred in the early growing period (June to July), whereas the second peak occurred during the reproduction stage of rice plants in August (Figure 1). Most of CH_4_ emissions in the atmosphere were observed during the rice growing season, and were rarely observed during the faba bean growing season. 

### 3.2. Nitrous Oxide Emissions

**Figure 2:**
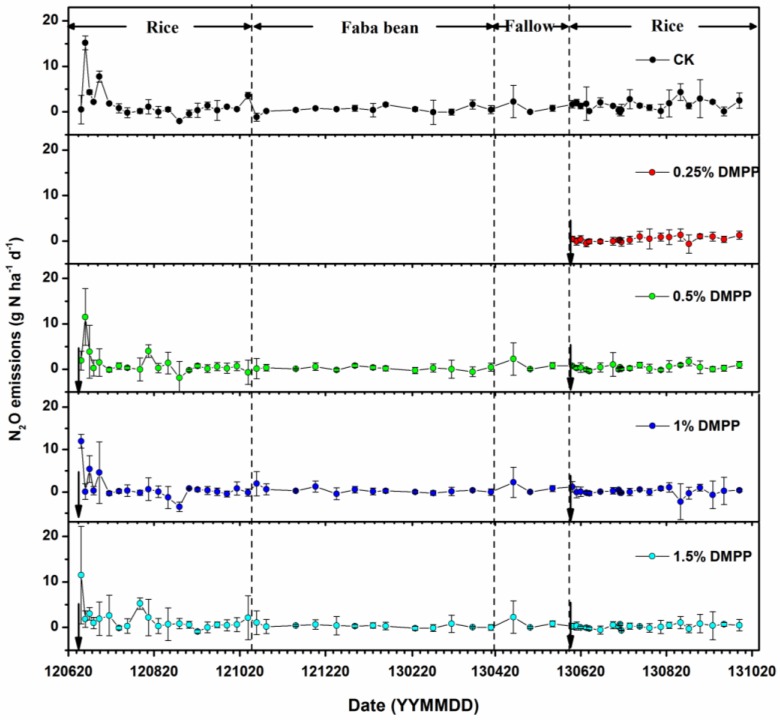
N_2_O emissions under different DMPP treatments.

For all of the treatments, a similar variable pattern of N_2_O emissions was observed throughout the year (Figure 2). Following the first week of flooding/fertilization, N_2_O was observed to decrease from 11.98 g N ha^−1^ d^−1^ to 0 g N ha^−1^ d^−1^, which quickly increased at the onset of the midseason aeration, and then just as quickly decreased. The N_2_O emissions decreased with the addition of DMPP during the entire season. All treatments with DMPP, particularly at the 1% level, demonstrated lower N_2_O emissions than the control. The cumulative N_2_O emissions at 0.25%, 0.5%, 1%, and 1.5% DMPP levels accounted for approximately 28.4%, 28.4%, 5.6%, and 15.2% of the CK treatments, respectively (Table 1).

More N_2_O was released into the ambient atmosphere from the paddy field during the rice growing season, in contrast with the faba bean growing season. The addition of DMPP resulted in lower levels of N_2_O emissions compared to the control (Table 1). 

### 3.3. Crop Yield

Under all treatments, the differences in the mean yields were not statistically significant (Table 2). Compared with CK, the treatments with 0.5% and 1% DMPP showed higher yields. GWPI indicated the yield-scale warming potential, as shown in Table 2. The GWPI was decreased by 33.3%, 56.9%, 47.1%, and 47.7% with the application of 0.25%, 0.5%, 1%, and 1.5% DMPP compared with the CK treatment, respectively.

### 3.4. Soil Inorganic N Concentration

**Figure 3:**
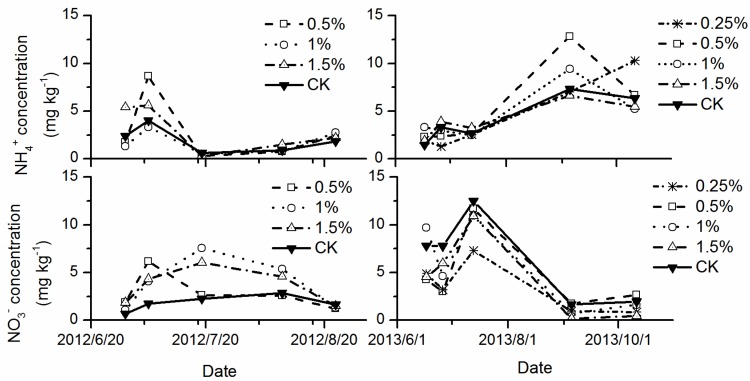
Variation of soil inorganic N concentration under different DMPP treatments.

Higher soil NH_4_^+^ concentrations existed under urea + DMPP treatments compared to the urea only treatment. Following the application of urea fertilizer, the soil NH_4_^+^ content decreased, and NO_3_^−^ increased more slowly with the DMPP application (Figure 3). In 2012, the mean soil NH_4_^+^ concentration was 2.79 mg kg^−1^, 1.75 mg kg^−1^, 3.00 mg kg^−1^, and 1.96 mg kg^−1^ for 0.5%, 1%, and 1.5% DMPP and CK treatments, respectively; the mean soil NO_3_^−^ concentration was 2.89 mg kg^−1^, 3.88 mg kg^−1^, 3.65 mg kg^−1^, and 1.84 mg kg^−1^ for the four treatments, respectively (Figure 3). In 2013, the mean soil ${\mathrm{NH}}_{4}^{+}$ concentration was 4.61 mg kg^−1^, 5.37 mg kg^−1^, 4.68 mg kg^−1^, 4.22 mg kg^−1^, and 4.22 mg kg^−1^ for 0.25%, 0.5%, 1%, and 1.5% DMPP and CK treatments, respectively; the mean soil NO_3_^−^ concentration was 3.44 mg kg^−1^, 4.67 mg kg^−1^, 5.53 mg kg^−1^, 4.41 mg kg^−1^, and 6.34 mg kg^−1^ (Figure 3).

CH_4_ emissions for all treatments had negative correlations with soil NH_4_^+^ concentrations, and positive correlations with soil NO_3_^−^ concentrations (Table 3). There was no significant relationship between N_2_O emissions and soil inorganic N concentrations. 

## 4. Discussion

### 4.1. Seasonal Variation of CH_4_ and N_2_O Emissions

Our results indicated that there was an evident variation in the CH_4_ and N_2_O emissions from paddy fields during the rice growing season in contrast to the faba bean growing season, with higher emission rates compared to those reported in previous studies. For instance, the highest and lowest CH_4_ emission values observed in Japan were 4.25 and 0.0062 kg C ha^−1^ day^−1^, respectively [26]. Other research [27] showed that CH_4_ emissions ranged from 0.17 kg C ha^−1^ day^−1^ to 0.63 kg C ha^−1^ day^−1^ during the rice growing season in Hubei Province, China. With three rotations, the relatively lower CH_4_ emission, which occurred during the non-rice periods, accounted for 16–49% of the total annual emissions [28]. Nitrous dioxide emissions from dry farmlands or paddy fields varied from 0.0017 g N ha^−1^ day^−1^ to 0.0296 g N ha^−1^ day^−1^ during the upland crop season, and the net average of N_2_O emissions during the rice growing season was 0.0119 g N ha^−1^ day^−1^ [29]. For paddy fields, 25–39% of N_2_O was generated during the rice growing season, with the remainder being formed during the off season [30,31].

Following the harvesting of rice, paddy fields serve as a minor source of CH_4_, which contribute only ~1% of the total CH_4_ emissions during the rice growing season. The fields become a significant source of N_2_O, accounting for 40–50% of annual emissions [32]. 

### 4.2. Inhibition of DMPP on CH_4_ and N_2_O Emissions

Our results clearly showed that DMPP substantially inhibited CH_4_ and N_2_O emissions, with reductions of 34–54% and 39–94%, respectively, compared with the control treatment during the rice growing season. According to a meta-analysis (111 records from 39 studies), DMPP is effective in reducing N_2_O emissions, with the highest inhibitory effect of 40% across all land-types, and 27% in paddy fields [33]. These data suggest that the application of DMPP in paddy fields is a feasible way to reduce GHG emissions, while enhancing the efficiency of nitrogen fertilizers. The basic mechanism is that DMPP can not only inhibit the first step of nitrification, but also can slow down the rate of NH_4_^+^ oxidation, and delay the transformation of NH_4_^+^ to NO_3_^−^ in the soil. This is because DMPP can repress the activities of *Nitrosomonas* bacteria [13,34] and inhibit the growth of ammonium-oxidizing bacteria (AOB) and ammonium-oxidizing archaea (AOA) [35,36,37,38]. 

In flooding paddy fields, oxygen is present at the floodwater/surface soil interlayer and in the rice rhizosphere [39,40]. In these areas, N_2_O emissions may be observed via the nitrification of ammonium and the denitrification of accumulated nitrate subsequent to the application of nitrogen fertilizers (e.g., urea) [41,42,43,44]. The NI (DMPP) used in this study demonstrated some specific effects on nitrogen-molecule transformation, and thus influenced the GHG emissions. 

Numerous controversial reports regarding the effects of nitrification-inhibited CH_4_ emissions exist. CH_4_ emissions caused by DMPP treatments are significantly lower than that caused by the treatment without DMPP, which may be attributed to the significant effect of DMPP on CH_4_ oxidation [19]. However, some reports revealed that DMPP exerted no obvious effect on CH_4_ emissions [16]. Another study observed that DMPP positively influenced the reduction of CH_4_ emissions [14,45]. In this study, lower emissions of CH_4_ occurred after treatment with DMPP + urea, compared with that observed with the urea treatment alone. 

We observed the impact of DMPP on CH_4_ emissions in paddy fields and found a significant reduction in emissions. The application of urea may promote ${\mathrm{NH}}_{4}^{+}$ and ${\mathrm{NO}}_{3}^{-}$ content in irrigated rice paddies. In this study, soil ${\mathrm{NH}}_{4}^{+}$ content decreased, whereas ${\mathrm{NO}}_{3}^{-}$ increased more slowly with the application of DMPP, with similar results under the application of NIs (e.g., dicyandiamide, neem, and nimin) plus urea, compared with those observed with the application of urea alone [11,46]. It is likely that ${\mathrm{NH}}_{4}^{+}$ inhibited the emission of CH_4_ and elevated CH_4_ oxidation caused by fertilization [6,47]. In this study, inorganic soil N concentration had a strong relationship with CH_4_ emissions, particularly a negative relationship for ${\mathrm{NH}}_{4}^{+}$ concentrations and CH_4_ emissions, and a positive relationship for ${\mathrm{NO}}_{3}^{-}$ concentrations and CH_4_ emissions. Urea acts as an electron donor that increases the methanotrophic microbial population, while simulating the oxidation of CH_4_ [48]. As observed in the present study, Bodelier [47,49] indicated that ${\mathrm{NH}}_{4}^{+}$-promoting methane oxidation dominated the rice ecosystem. 

### 4.3. Optimal Quantity of DMPP Application

Our results suggested that there was maximal reduction in CH_4_ (39%) and N_2_O (34%) emissions with 0.5% and 1% DMPP of nitrogen fertilizers in the paddy fields. Compared with other experiments, there was an evident variation in the quantity of DMPP applied with regard to soil type, climate conditions, crops, reduction of CH_4_ and N_2_O, etc. For instance, the application of DMPP with 0.5% urea-N in greenhouse vegetable soils significantly reduced N_2_O emissions and acted to delay ammonia oxidation [50]. Within a specific temperature range (5–35 °C), DMPP with 0.39% urea-N (1.84 kg t^−1^ urea) had the capacity to inhibit N_2_O emissions, with a 14–76% reduction in pasture soils and a 19–99% reduction in Pin Gin, Mackay, and Dookie soils; the effectiveness in reduction of the experiments decreased with increasing temperatures [51]. With 0.42% nitrogen fertilizer as an active ingredient (3 mg kg^−1^ soil DMPP with 715 mg N kg^−1^ soil), DMPP was observed to slow ${\mathrm{NH}}_{4}^{+}$ oxidation considerably, and reduced N_2_O emissions by 83–95% under both 40% and 60% WFPS (water-filled porespace) [18]. The addition of 1% DMPP decreased the cumulative N_2_O emissions of soils by 73.4% [52]. DMPP with mineral fertilizers, and at a low concentration of 1%, specifically inhibited nitration and stabilized ${\mathrm{NH}}_{4}^{+}$ for several weeks [13]. Thus, the optimal quantity of DMPP application is contingent on soil type, crops, fertilizers, and climate.

## 5. Conclusions

Our results indicated that the application of DMPP with nitrogen fertilizers is a feasible way to reduce N_2_O and CH_4_ emissions in paddy fields, where the effectiveness of the reduction is contingent on the levels of DMPP that are applied. The 0.5–1% DMPP nitrogen fertilizer was found to be optimal in consideration of the reduction of CH_4_ and N_2_O emissions, as well as GWPI. These results have important implications in agricultural management as a strategy to mitigate GHG emissions.

## Figures and Tables

**Table 1:** Seasonal cumulative CH_4_ and N_2_O emissions under different treatments in the paddy fields in 2012 and 2013.

Treatments	Rice Growing Season				Faba Bean Growing Season	Fallow Season
	2012	Reduction (%)	2013	Reduction (%)	2012–2013	Flooded
CH_4_ emissions (kg C ha^−1^)						
0.25% DMPP	-	-	318.23 ± 6.50 b	33.5	-	-
0.5% DMPP	250.08 ± 3.64 c	46.2	220.26 ± 6.52 e	53.9	−0.24 ± 0.07 c	-
1% DMPP	246.96 ± 4.36 c	46.9	271.69 ± 8.27 c	43.7	0.07 ± 0.06 b	-
1.5% DMPP	297.18 ± 6.06 b	36.1	253.57 ± 6.49 d	47.0	−0.22 ± 0.06 c	-
CK	464.97 ± 8.36 a	-	478.23 ± 6.05 a	-	0.49 ± 0.07 a	101.51 ± 4.35
N_2_O emissions (kg N ha^−1^)						
0.25% DMPP	-	-	0.056 ± 0.006 b	71.6	-	-
0.5% DMPP	0.101 ± 0.01 b	38.8	0.056 ± 0.005 b	71.6	0.025 ± 0.01 c	-
1% DMPP	0.050 ± 0.009 c	69.7	0.011 ± 0.007 d	94.4	0.052 ± 0.01 b	-
1.5% DMPP	0.059 ± 0.012 a	64.2	0.030 ± 0.006 c	84.8	0.050 ± 0.012 b	-
CK	0.165 ± 0.005 a	-	0.197 ± 0.008 a	-	0.099 ± 0.009 a	0.022 ± 0.011

Note: Rice growing seasons were from 29 June to 26 October and 14 June to 11 October in 2012 and 2013, respectively. The faba bean growing season was from 1 November 2012 to 17 April 2013. After the faba beans were harvested, the field was flooded from 3 May to 31 May 2013. The different letters represent a significant difference (*p* < 0.05) among all the treatments. DMPP: 3,4-Dimethylpyrazole phosphate.

**Table 2:** Rice yield and GWPI under different treatments in the paddy fields in 2012 and 2013.

Treatments	Crop yields (kg ha^−1^)		GWP (kg CO_2_-eq ha^−1^)		GWPI (kg CO_2_-eq kg^−1^ yield)	
	2012	2013	2012	2013	2012	2013
0.25% DMPP	-	8764.24 ± 300.86 a	-	8937.72	-	1.020
0.5% DMPP	8825.00 ± 116.59 a	9365.13 ± 537.33 a	7051.44	6194.56	0.799	0.661
1% DMPP	9047.32 ± 378.76 a	9336.67 ± 288.95 a	6939.24	7612.68	0.767	0.815
1.5% DMPP	9002.43 ± 207.31 a	8880.77 ± 441.30 a	8349.78	7114.57	0.928	0.801
CK	8993.97 ± 100.03 a	8820.87 ± 254.56 a	13099.54	13486.41	1.456	1.529

Note: Mean ± standard error of three replicates is shown in the table. The different letters represent a significant difference (*p* < 0.05) among all the treatments. GWPI: The index of yield-scaled CO2-eq; GWP: The global warming potential.

**Table 3:** Pearson correlation between greenhouse gas emissions and soil inorganic N concentrations in the paddy fields.

	CH_4_	N_2_O	NH_4_^+^	NO_3_^−^
CH_4_	1	−0.154	−0.570 **	0.439 **
N_2_O		1	0.068	−0.161
${\mathrm{NH}}_{4}^{+}$			1	−0.323 *
${\mathrm{NO}}_{3}^{-}$				1

Note: ** Correlation is significant at the 0.01 level (two-tailed); * Correlation is significant at the 0.05 level (two-tailed).
